# Supplementary material for: Splice-Junction-Based Mapping of Alternative Isoforms in the Human Proteome
Source: Cell Rep. Author manuscript; Available in PMC 2020 Jan 15. (PMC6961840; doi:10.1016/j.celrep.2019.11.026)
Supplement: 2 [file NIHMS1546469-supplement-2.zip › DF1/PXD010154/Heart/Heart_80_ILKAP_GPLLFDDLPPASSTDSGTVFR.pdf]

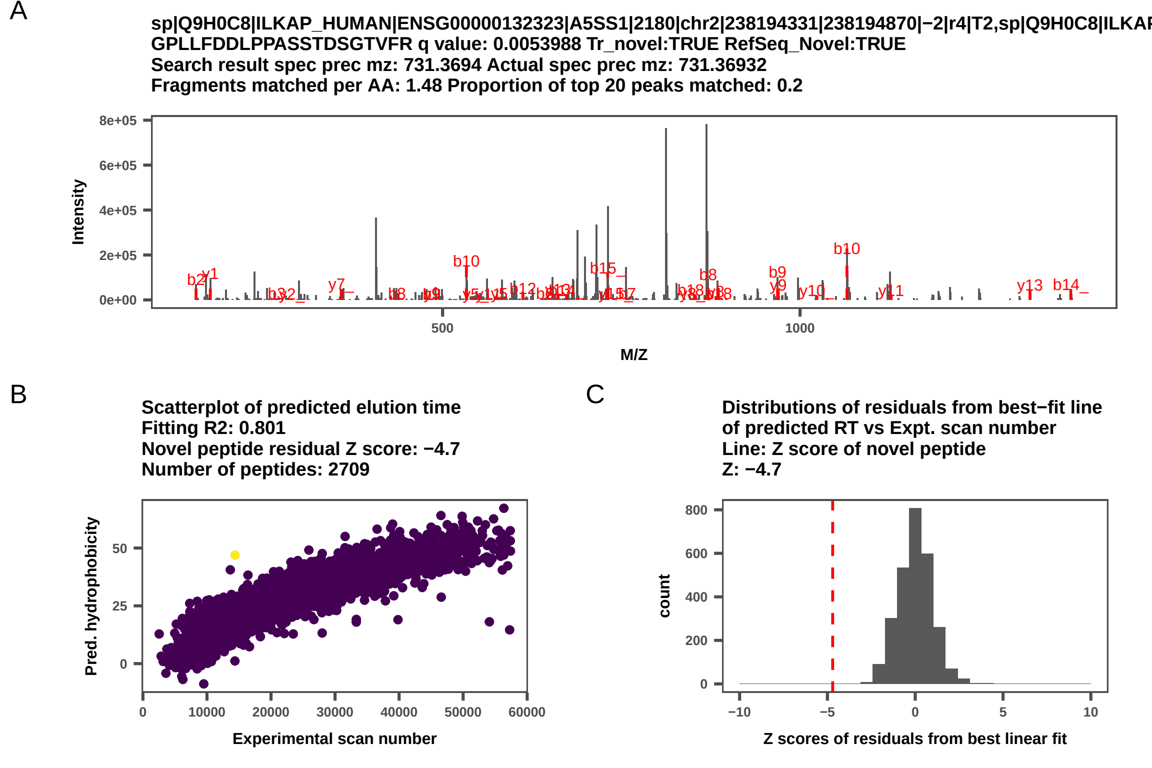
A
sp|Q9H0C8|ILKAP_HUMAN|ENSG00000132323|A5SS1|2180|chr2|238194331|238194870|−2|r4|T2,sp|Q9H0C8|ILKAP
GPLLFDDLPPASSTDSGTVFR q value: 0.0053988 Tr_novel:TRUE RefSeq_Novel:TRUE
Search result spec prec mz: 731.3694 Actual spec prec mz: 731.36932
Fragments matched per AA: 1.48 Proportion of top 20 peaks matched: 0.2
Intensity
8e+05
6e+05
4e+05
2e+05
0e+00
500
1000
M/Z
b10
b15_
b8
b9
y9
b10
y10_
y11
y13
b14_
B
Scatterplot of predicted elution time
Fitting R2: 0.801
Novel peptide residual Z score: −4.7
Number of peptides: 2709
Pred. hydrophobicity
50
25
0
0
10000
20000
30000
40000
50000
60000
Experimental scan number
C
Distributions of residuals from best−fit line
of predicted RT vs Expt. scan number
Line: Z score of novel peptide
Z: −4.7
count
800
600
400
200
0
−10
−5
0
5
10
Z scores of residuals from best linear fit
